# Supplementary material for: Biological Activity of NHC-Gold-Alkynyl Complexes Derived from 3-Hydroxyflavones
Source: Pharmaceutics. 2022 Sep 27;14(10):2064. doi: 10.3390/pharmaceutics14102064 (PMC9612383; doi:10.3390/pharmaceutics14102064)
Supplement: Supplementary file 1 [file pharmaceutics-14-02064-s001.zip › pharmaceutics-1921963-supplementary.pdf]

Biological Activity of NHC-Gold-Alkyne Complexes Derived from 3-hydroxyflavones

Inés Mármol [1,2], Javier Quero [2], Paula Azcárate [1], Elena Atrián-Blasco [3], Carla Ramos [4], Joana Santos [4], María Concepción Gimeno [1], María Jesús Rodríguez-Yoldi [2,*] and Elena Cerrada [1,*]

[1] Departamento de Química Inorgánica, Instituto de Síntesis Química y Catálisis Homogénea-ISQCH, Universidad de Zaragoza-C.S.I.C., Pedro Cerbuna 12, 50009 Zaragoza, Spain
[2] Departamento de Farmacología y Fisiología, Medicina Legal y Forense, Unidad de Fisiología, Universidad de Zaragoza, CIBERobn, IIS Aragón, IA2, 50013 Zaragoza, Spain
[3] Instituto de Nanociencia y Materiales de Aragón (INMA), CSIC-Universidad de Zaragoza, 50009 Zaragoza, Spain
[4] Escola Superior de Tecnologia e Gestão, Instituto Politécnico de Viana do Castelo, Avenida do Atlântico No. 644, 4900-348 Viana do Castelo, Portugal
* Correspondence: mjrodyol@unizar.es (M.J.R.-Y.); ecerrada@unizar.es (E.C.)

Table of contents:

Figures S1–S16. ^{1}H NMR and $^{13}C\{^{1}H\}$ NMR spectra

Figure S17. UV-Vis spectra of gold complexes recorded at different times under physiological conditions.

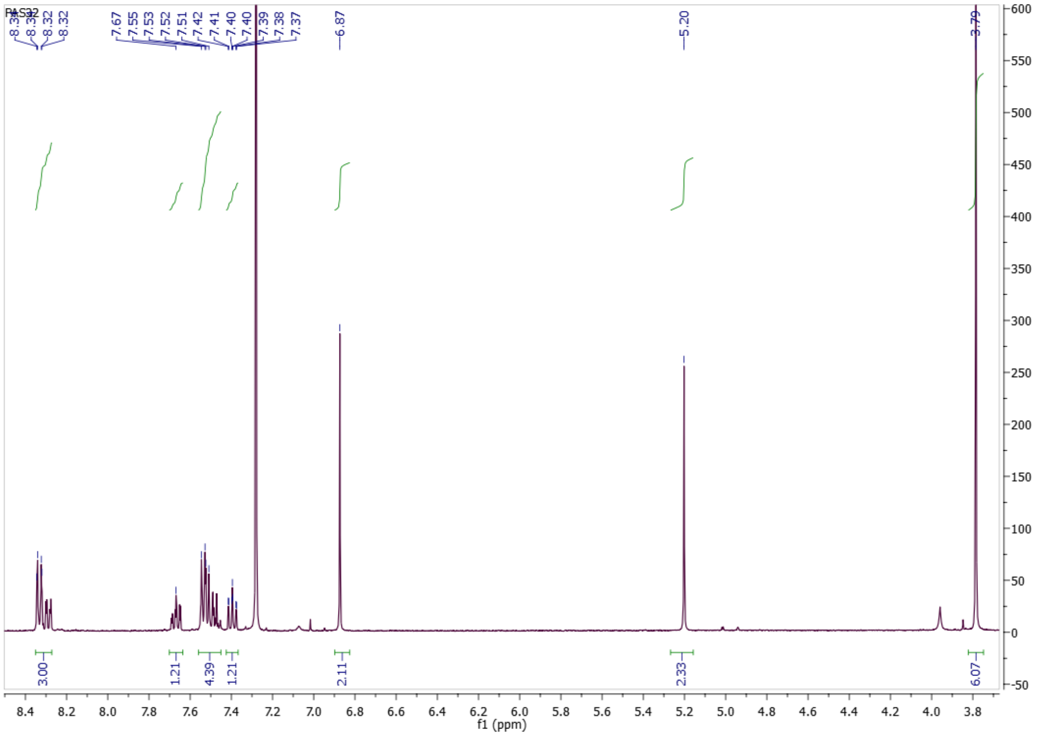

Figure S1. ^{1}H NMR spectrum of *[Au(La)(IMe)]* (R = H) (**1**) in $CDCl_3$

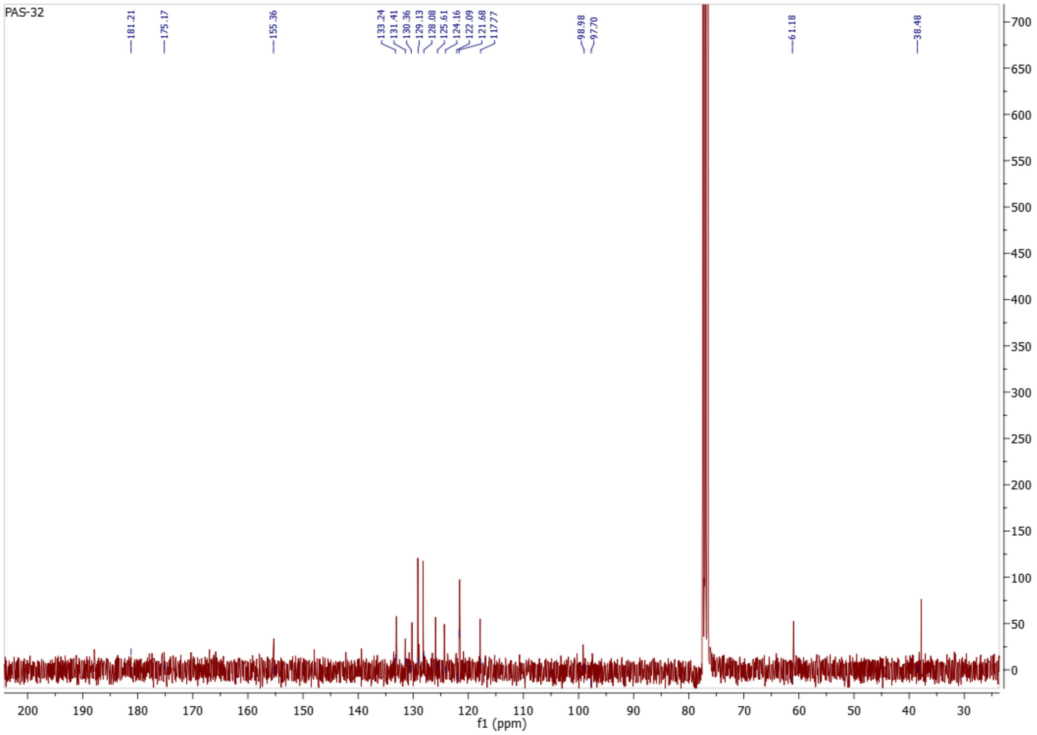

Figure S2. $^{13}C\{^{1}H\}$ NMR spectrum of *[Au(La)(IMe)]* (R = H) (**1**) in $CDCl_3$

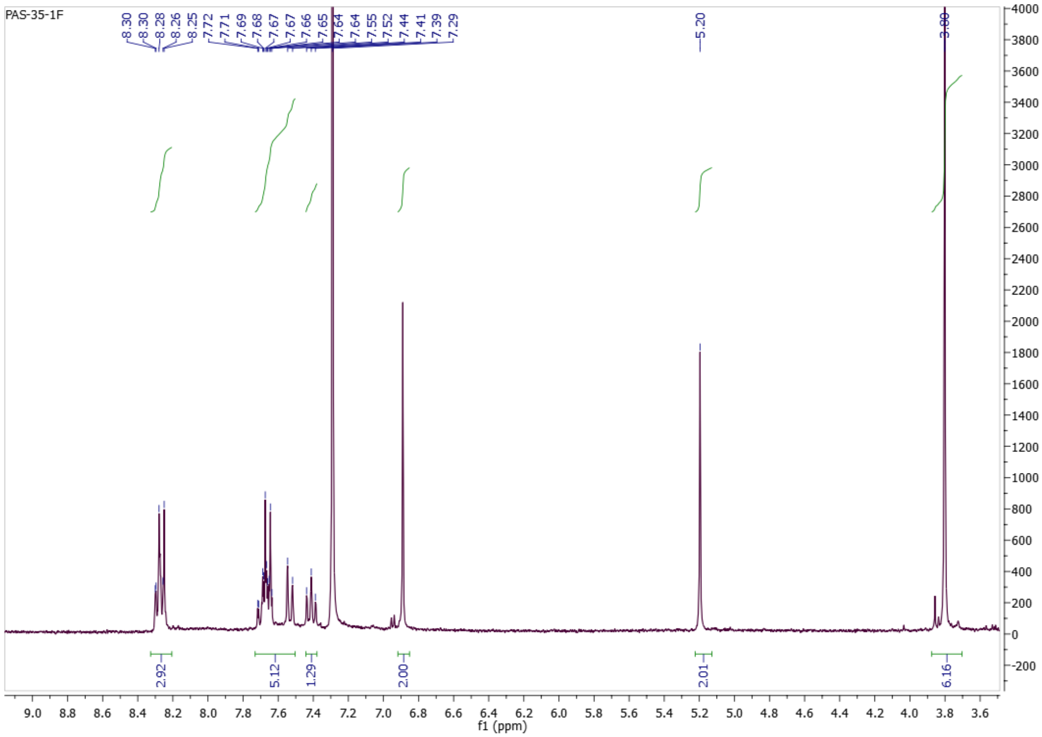

Figure S3. ^{1}H NMR spectrum of *[Au(Lb)(IMe)]* (R = Br) (**2**) in $CDCl_3$

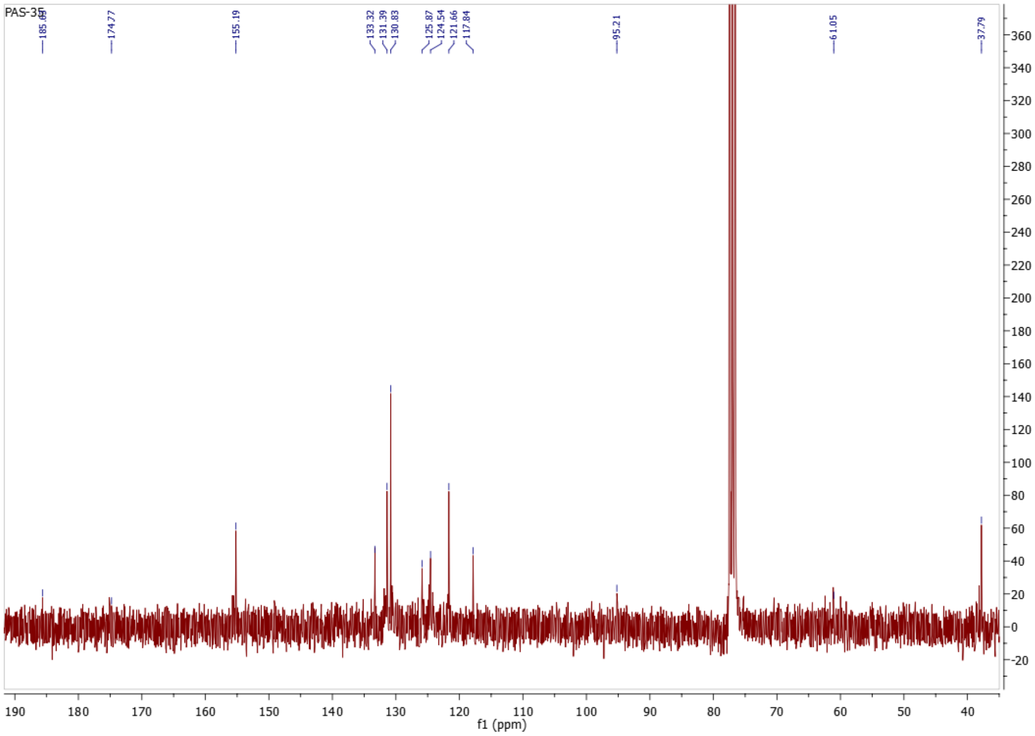

Figure S4. $^{13}C\{^{1}H\}$ NMR spectrum of *[Au(Lb)(IMe)]* (R = Br) (**2**) in $CDCl_3$

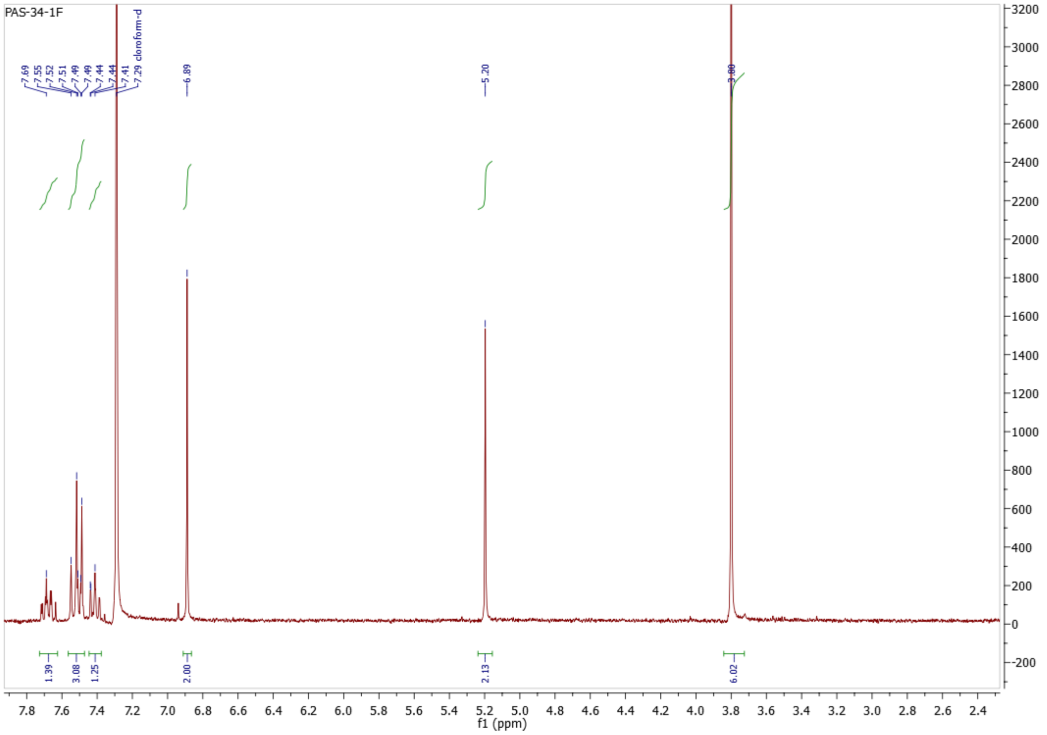

Figure S5. ^{1}H NMR spectrum of *[Au(Lc)(IMe)]* (R = Cl) (**3**) in $CDCl_3$

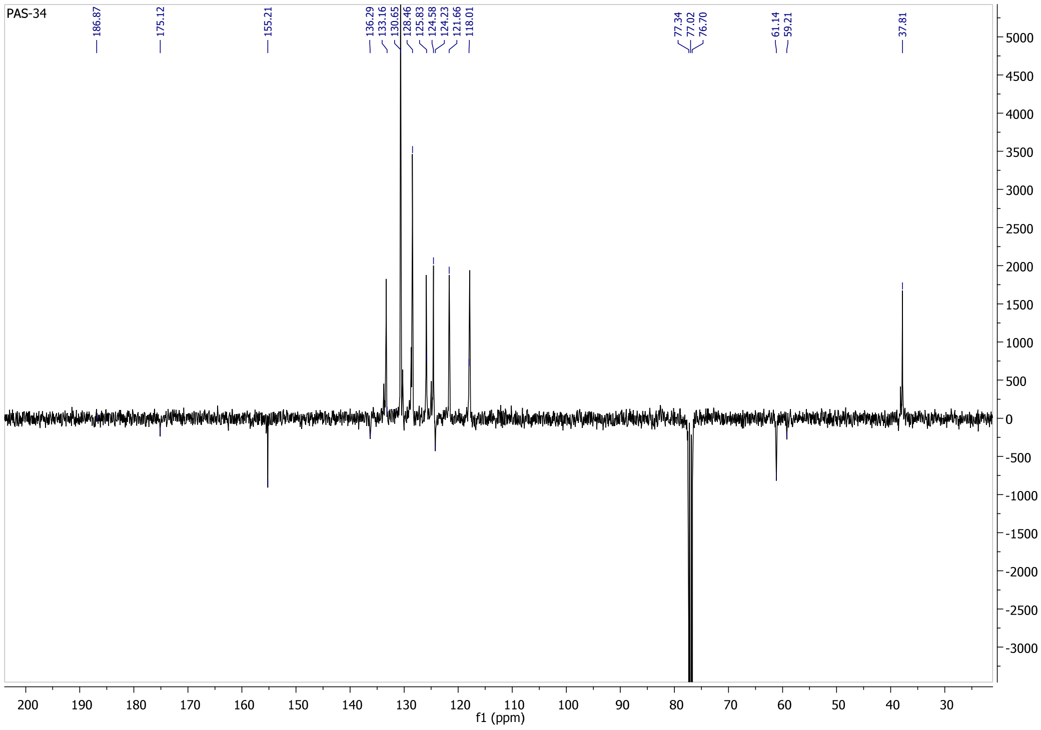

Figure S6. $^{13}C\{^1H\}$-apt NMR spectrum of *[Au(Lc)(IMe)]* (R = Cl) (**3**) in $CDCl_3$

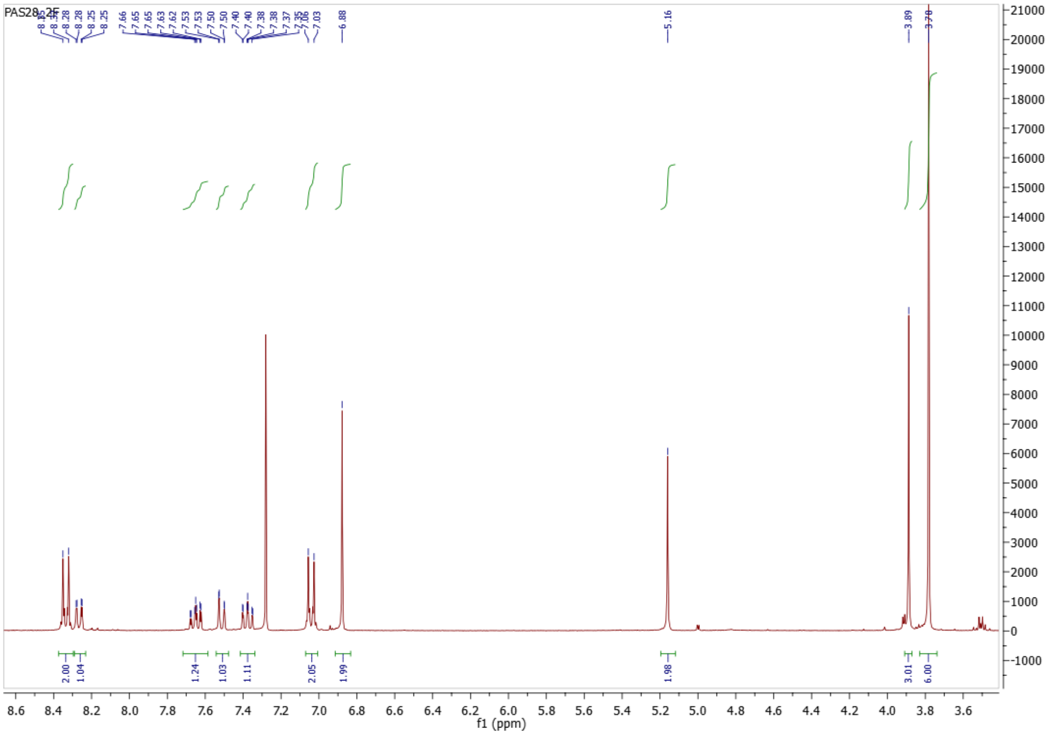

Figure S7. ^{1}H NMR spectrum of *[Au(Ld)(IMe)]* (R = OMe) (**4**) in $CDCl_3$

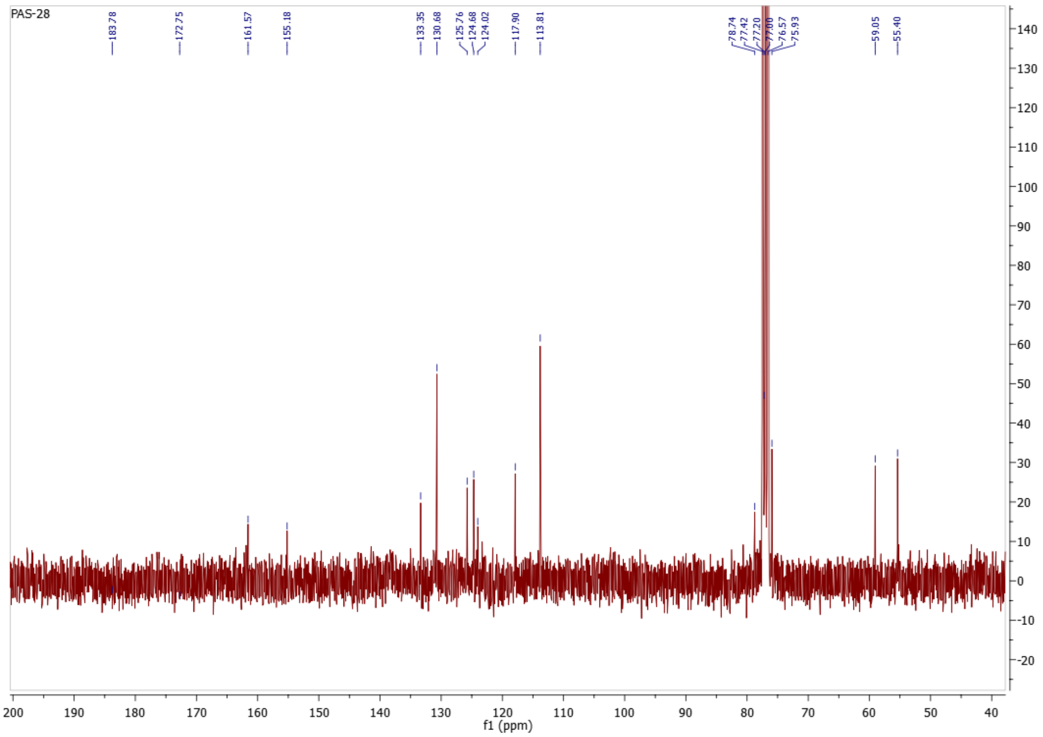

Figure S8. $^{13}C\{^{1}H\}$ NMR spectrum of *[Au(Ld)(IMe)]* (R = OMe) (**4**) in $CDCl_3$

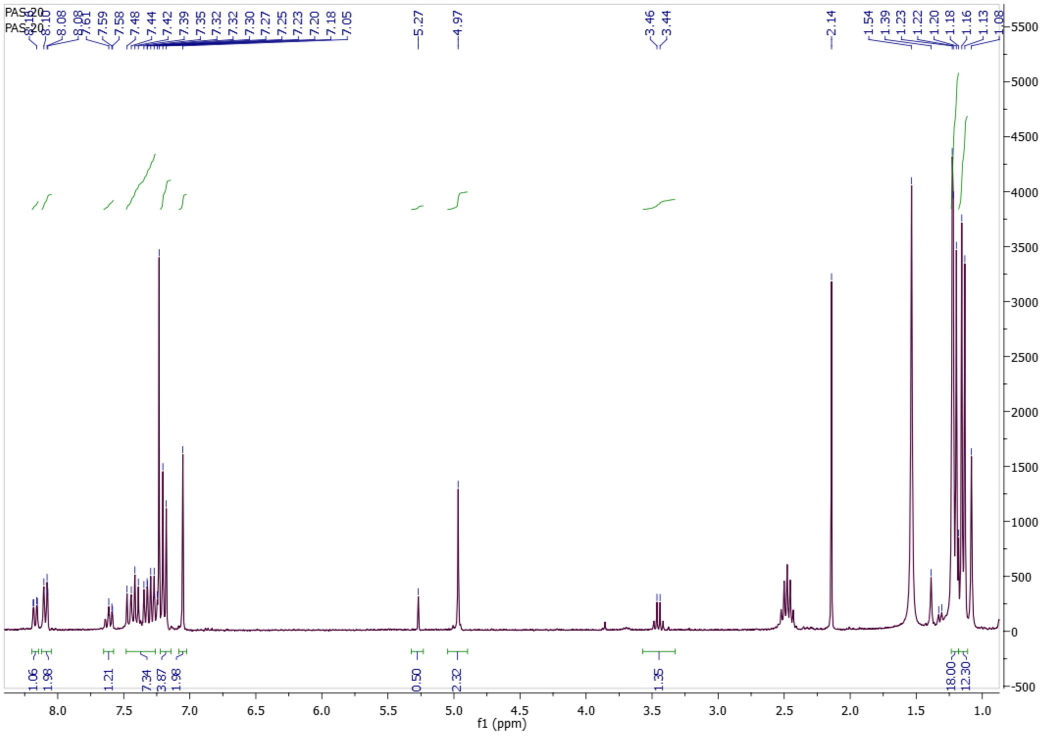

Figure S9. ^{1}H NMR spectrum of *[Au(La)(IPr)]* (R = H) (**5**) in $CDCl_3$

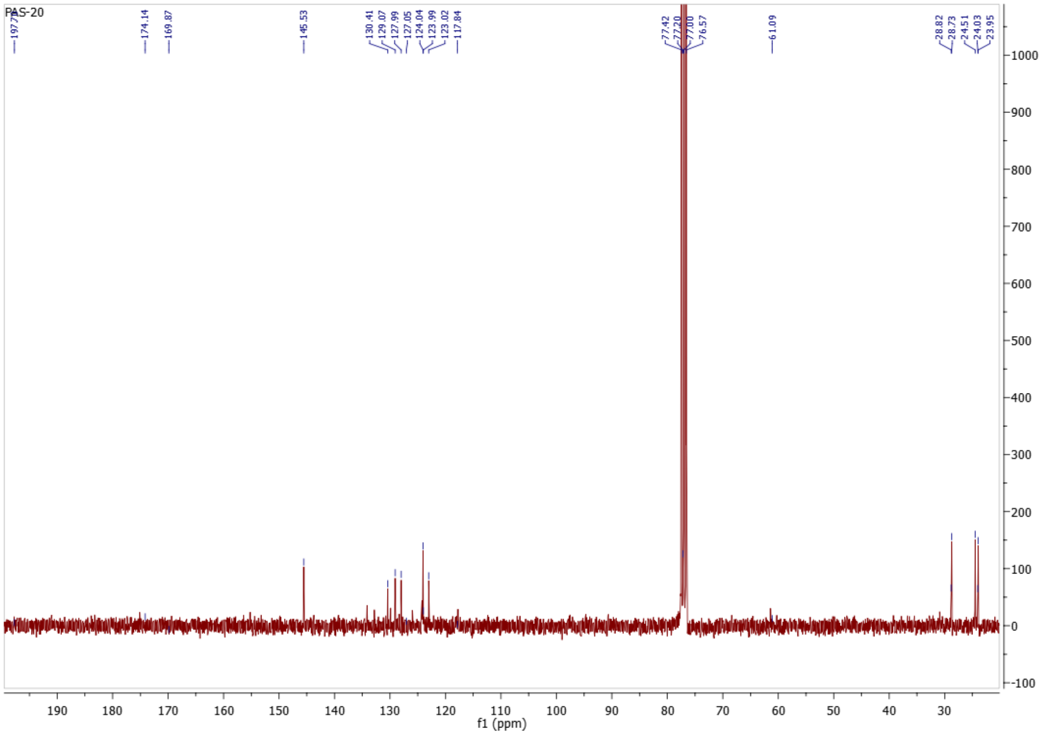

Figure S10. $^{13}C\{^{1}H\}$ NMR spectrum of *[Au(La)(IPr)]* (R = H) (**5**) in $CDCl_3$

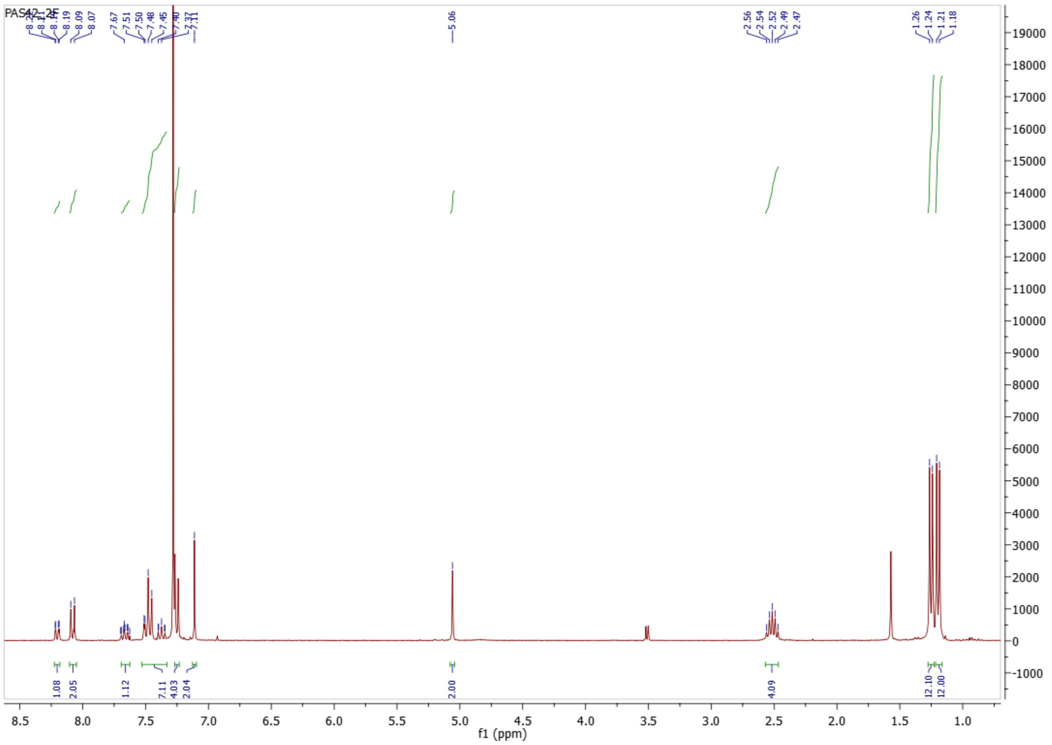

Figure S11. ^{1}H NMR spectrum of *[Au(Lb)(IPr)]* (R = Br) (**6**) in $CDCl_3$

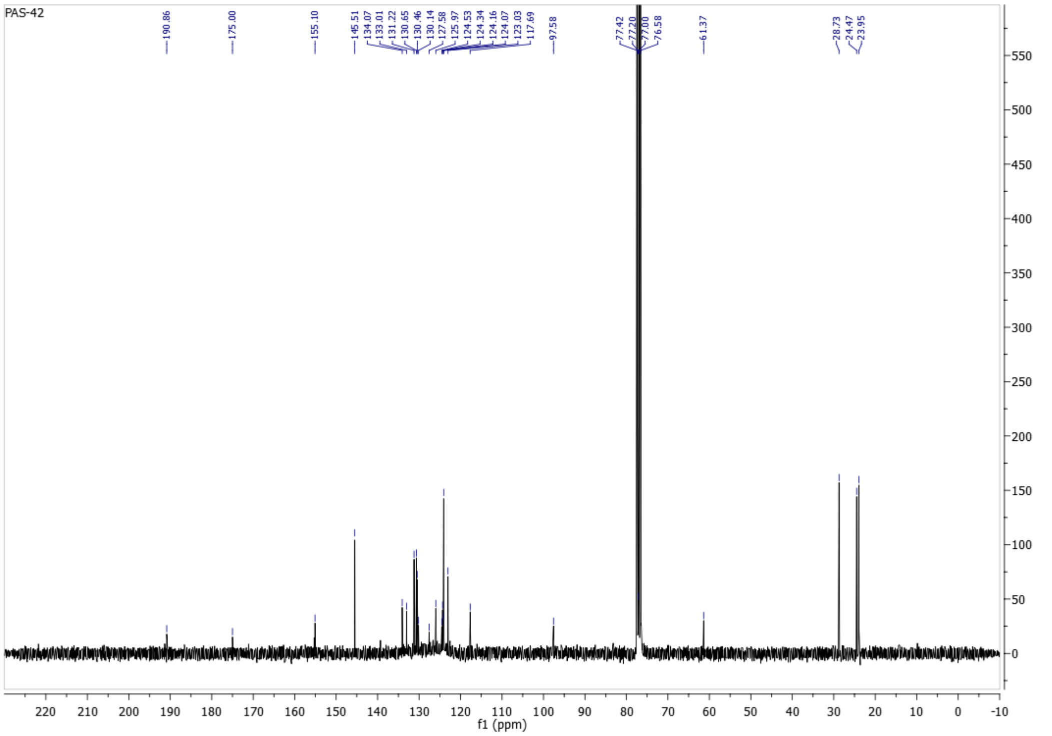

Figure S12. $^{13}C\{^{1}H\}$ NMR spectrum of *[Au(Lb)(IPr)]* (R = Br) (**6**) in $CDCl_3$

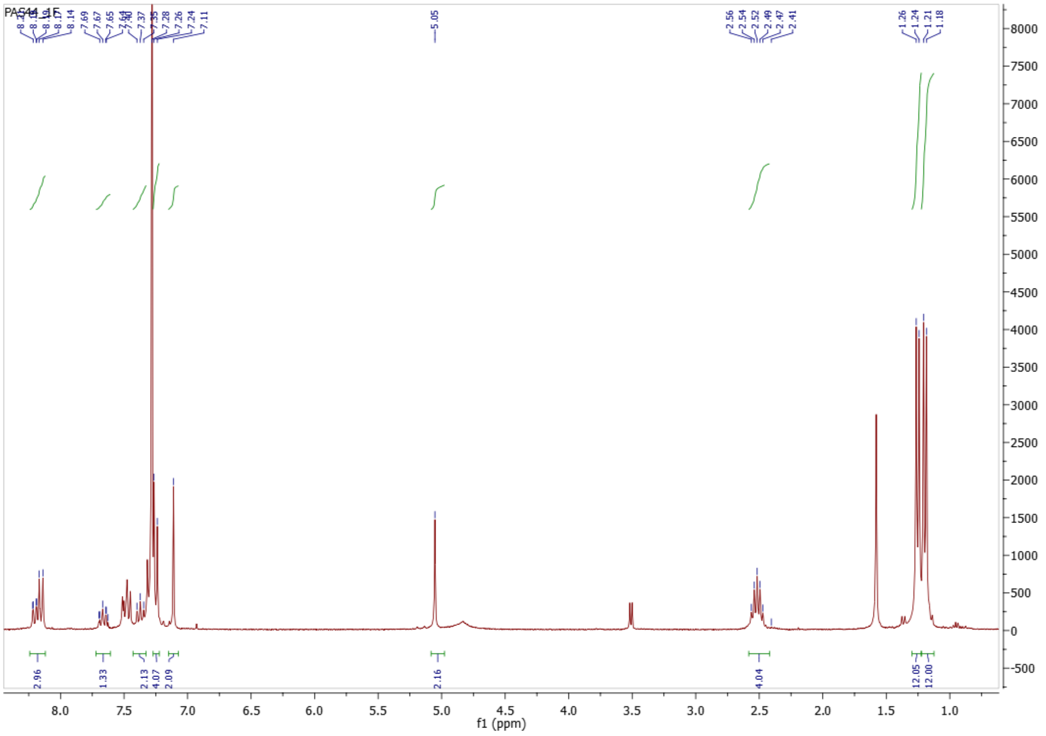

Figure S13. ^{1}H NMR spectrum of *[Au(Lc)(IPr)]* (R = Cl) (**7**) in $CDCl_3$

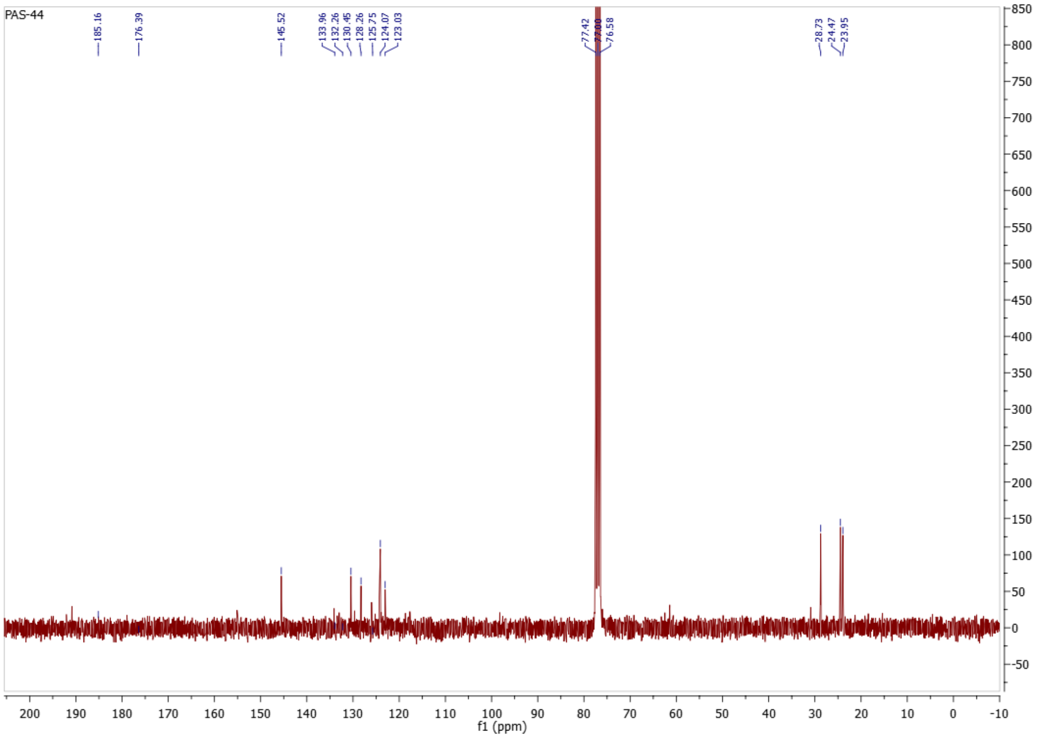

Figure S14. $^{13}C\{^{1}H\}$ NMR spectrum of *[Au(Lc)(IPr)]* (R = Cl) (**7**) in $CDCl_3$

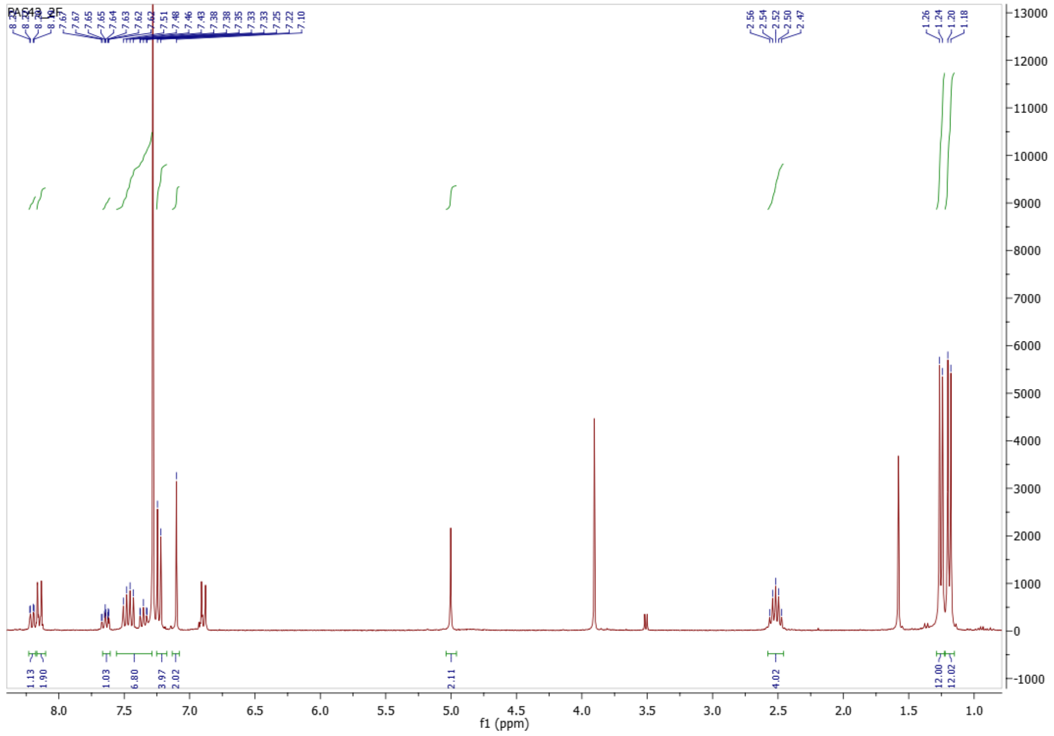

Figure S15. ^{1}H NMR spectrum of *[Au(Ld)(IPr)]* (R = OMe) (**8**) in $CDCl_3$

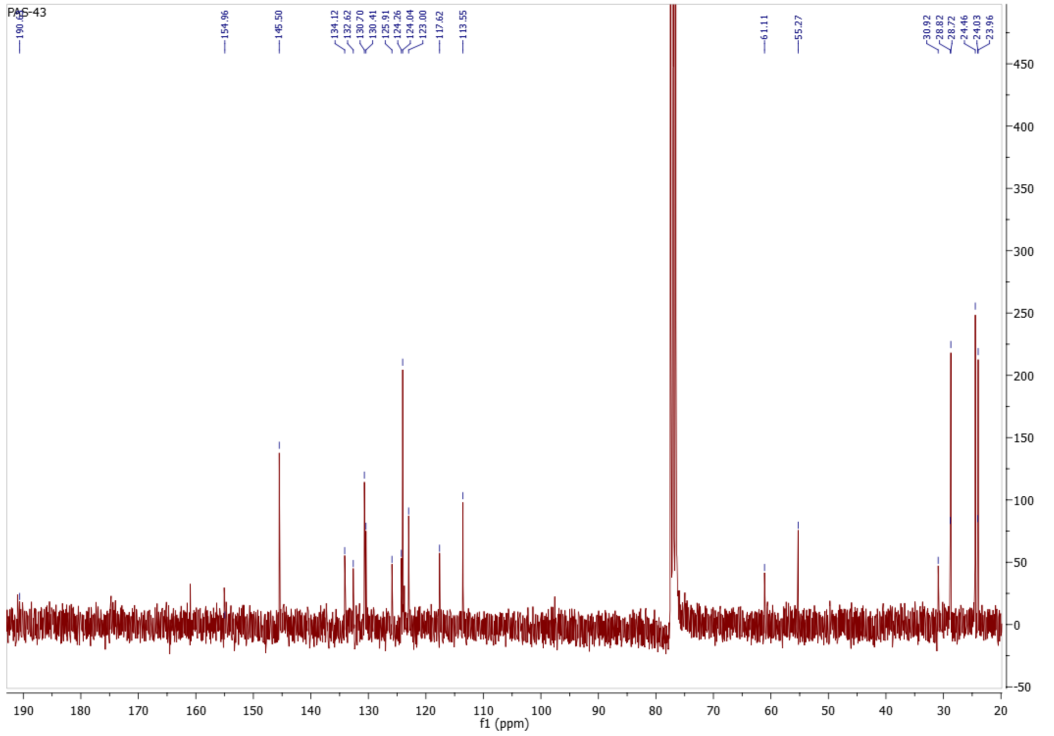

Figure S16. $^{13}C\{^{1}H\}$ NMR spectrum of *[Au(Ld)(IPr)]* (R = OMe) (**8**) in $CDCl_3$

Figure S17. UV-Vis spectra of gold complexes recorded at different times for 24 h to test their stability under physiological conditions. [complex] = $3 \cdot 10^{-5}$M diluted in PBS at 37 ºC.

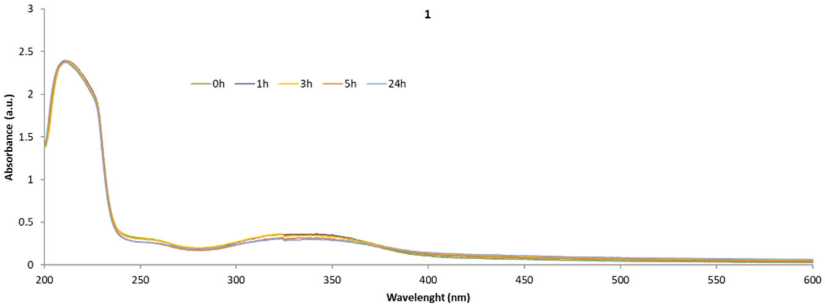

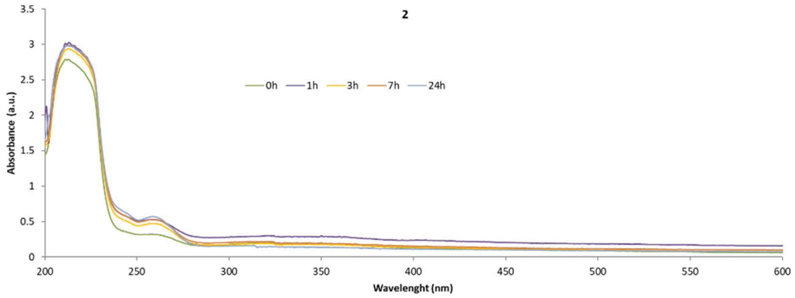

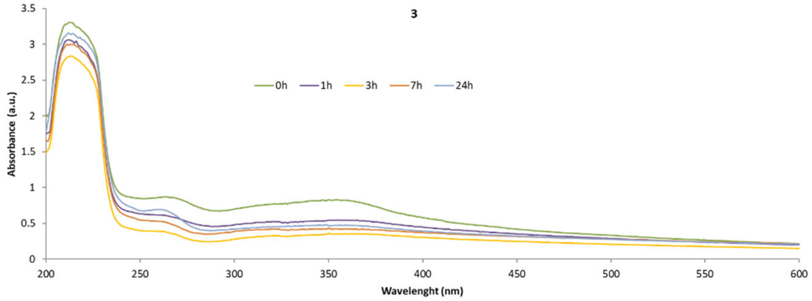

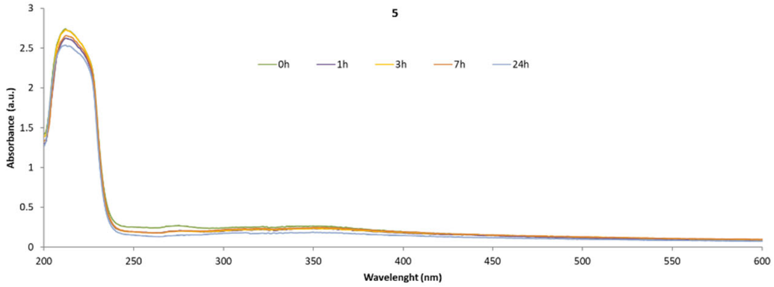

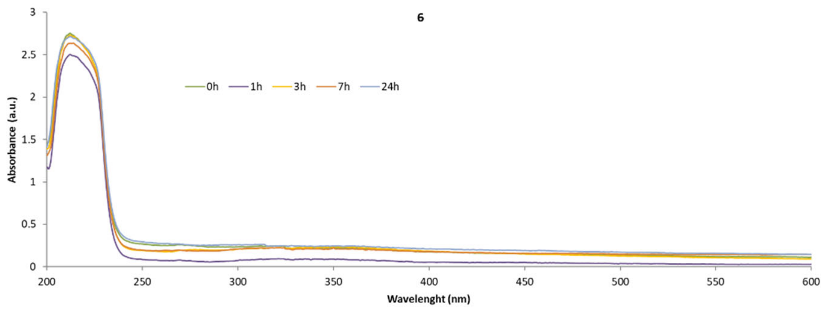
6
0h
1h
3h
7h
24h
Absorbance (a.u.)
Wavelenght (nm)
3
2.5
2
1.5
1
0.5
0
200
250
300
350
400
450
500
550
600

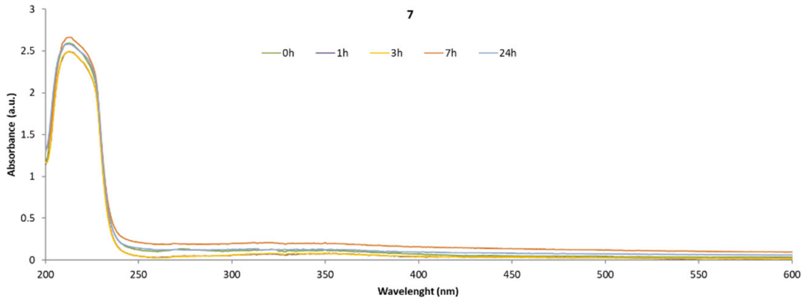
7
0h
1h
3h
7h
24h
Absorbance (a.u.)
Wavelenght (nm)
3
2.5
2
1.5
1
0.5
0
200
250
300
350
400
450
500
550
600

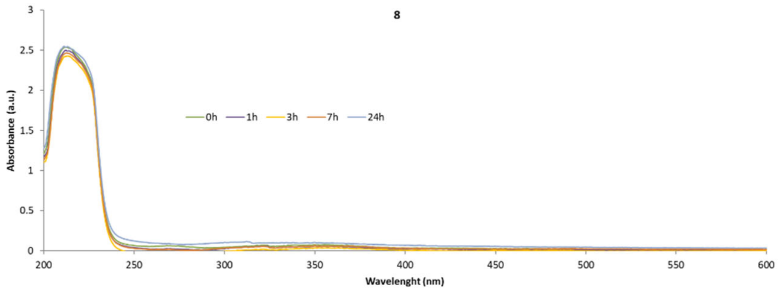
8
0h
1h
3h
7h
24h
Absorbance (a.u.)
Wavelenght (nm)
3
2.5
2
1.5
1
0.5
0
200
250
300
350
400
450
500
550
600
